# Telemedicine Versus Standard Follow-Up Care for Diabetes-Related Foot Ulcers: Protocol for a Cluster Randomized Controlled Noninferiority Trial (DiaFOTo)

**DOI:** 10.2196/resprot.5646

**Published:** 2016-07-18

**Authors:** Marjolein M Iversen, Birgitte Espehaug, Marie F Hausken, Marit Graue, Truls Østbye, Svein Skeie, John G Cooper, Grethe S Tell, Bodo Erhardt Günther, Håvard Dale, Hilde Smith-Strøm, Beate-Christin H Kolltveit, Marit Kirkevold, Berit Rokne

**Affiliations:** ^1^ Centre for Evidence-Based Practice Faculty of Health and Social Sciences Bergen University College Bergen Norway; ^2^ Section of Endocrinology Department of Medicine Stavanger University Hospital Stavanger Norway; ^3^ Duke Global Health Institute Duke University Durham, NC United States; ^4^ Department of Global Public Health and Primary Care University of Bergen Bergen Norway; ^5^ Department of Surgery Stord General Hospital Stord Norway; ^6^ Department of Orthopedic Surgery Haukeland University Hospital Bergen Norway; ^7^ Institute of Health and Society Faculty of Medicine University of Oslo Oslo Norway

**Keywords:** diabetes, diabetic foot, foot ulcer, telemedicine, randomized controlled trial, primary care, delivery of health care, integrated, complex intervention, patient-reported outcomes, Norway, cluster RCT

## Abstract

**Background:**

This paper presents the protocol for an ongoing study to evaluate a telemedicine follow-up intervention for patients with diabetes-related foot ulcers. Diabetes-related foot ulcers represent challenges for patients and the health services. The large increase in the prevalence of diabetes, combined with the aging population, means that the absolute number of patients with diabetes-related foot ulcers is likely to continue to increase. Health care services therefore need to provide close clinical follow-up care for people with diabetes both in primary and specialist care. Information and communication technologies may enable more integrated treatment and care pathways across organizational boundaries. However, we lack knowledge about the effect of telemedicine follow-up and how such services can be optimally organized.

**Objective:**

To present the design and methods of a study evaluating a telemedicine follow-up intervention for patients with diabetes-related foot ulcers.

**Methods:**

The study is designed as a cluster randomized controlled trial (noninferiority trial) involving municipalities or municipality districts (clusters) belonging to one clinical site in Western Norway. The study includes patients with type 1 and type 2 diabetes presenting with a new foot ulcer at the initial visit to the clinic. Patients in the intervention group receive telemedicine follow-up care in the community. The key ingredient in the intervention is the close integration between health care levels. The intervention is facilitated by the use of an interactive wound platform consisting of a Web-based ulcer record combined with a mobile phone, enabling counseling and communication between nurses in the community and specialist health care. Patients in the control group receive standard hospital outpatient care. The primary endpoint in the trial is healing time; secondary outcomes include amputation and death, patient-reported outcome measures, and follow-up data on the recurrence of foot ulcers. In addition, qualitative substudies are being performed to provide a more comprehensive evaluation of the ongoing processes during the trial with the patients in the intervention and control groups and those health care professionals either working in primary care or in specialist care delivering the intervention.

**Results:**

The project has been funded. The inclusion of patients started in September 2012. Because recruitment goals were not met in the initial period, two more clinical sites have been included to meet sample size requirements. Patient recruitment will continue until June 2016. Data collection in the qualitative substudies has been completed.

**Conclusions:**

This telemedicine trial operates in a novel setting and targets patients with diabetes-related foot ulcers during a 12-month follow-up period. The trial addresses whether integrated care using telemedicine between primary and specialist health care can be an equivalent alternative to standard outpatient care.

**Trial Registration:**

ClinicalTrials.gov NCT01710774; https://clinicaltrials.gov/ct2/show/NCT01710774 (Archived by WebCite at http://www.webcitation.org/6im6KfFov).

## Introduction

### Background

The prevalence of diabetes is expected to increase both in Norway and globally [1]. In Norway, about 200,000 people have been diagnosed with diabetes [2] with an annual increase of 8000 to 10,000 forecast. In addition, an estimated 150,000 people have undiagnosed type 2 diabetes [2,3]. The increasing prevalence of diabetes, especially type 2 diabetes, combined with an increasing proportion of older people in the population present great challenges for the health care services [2,4,5]. An epidemiologic study of diabetes-related foot ulcers among community-dwelling adults and older people based on data from the Nord-Trøndelag Health Study (HUNT2) showed that a history of foot ulcer was significantly associated with increased mortality and that about 10% of people with diabetes reported a history of foot ulcer [6-8]. Other studies have shown that a foot ulcer is associated with reduced quality of life, social limitations, and pain [9]. The cost of treating foot ulcers is also considerable [10]. It is therefore important to start treatment early and have a close, well-organized follow-up for patients with diabetes-related foot ulcers to improve the management of the diabetic foot [11].

### State of the Evidence, Relevance, and Innovation Potential

The main goal for the Norwegian Coordination Reform in the health care sector in January 2012 was to obtain coordinated and integrated health care for patients, especially for those with complex conditions [12]. In this reform, electronic communication and use of telemedicine is emphasized. Qualitative studies of diabetes-related foot ulcers [13-15] have shown that using telemedicine can result in follow-up care of similar quality to standard outpatient care while at the same time enabling more flexible organization and greater patient satisfaction. Patients with diabetes foot ulcers are prone to adverse outcomes because of rapid deterioration of the ulcer or the onset of infection. To date, randomized controlled studies have not confirmed that telemedicine follow-up care for patients with diabetes-related foot ulcers results in equivalent healing time when compared with standard outpatient care in specialist health care [16,17]. Therefore, there is a need for such studies to document the safety and effectiveness of a telemedicine-based follow-up. This project is expected to increase the focus on research related to integrated care [3,12].

This paper presents the protocol for an ongoing cluster randomized controlled noninferiority trial, DiaFOTo (Diabetic Foot and Telemedical Images Project). The trial is designed to compare the effect of telemedicine follow-up in primary care to standard hospital outpatient care on ulcer healing time. In addition, qualitative data were collected to evaluate ongoing processes and further elaborate the experiences of patients and health care professionals during the intervention period. These qualitative studies are part of a larger program supported by the Norwegian Research Council (DiaHEALTH-221065/F40) to promote patient and professional competencies in diabetes care and management.

### Aims and Research Questions

The main aim of this trial is to evaluate whether follow-up of patients with diabetes-related foot ulcers in primary care, in collaboration with hospital outpatient specialist care, is noninferior to standard outpatient care in terms of ulcer healing time.

Our primary research question is whether healing time (within 12 months) of diabetes-related foot ulcers treated in primary care in collaboration with telemedicine consultations with a hospital outpatient is no worse than with standard hospital outpatient care. The corresponding null hypothesis: mean difference in healing time is 1.5 months or less for diabetes-related foot ulcers with telemedicine follow-up in primary care, compared to standard hospital outpatient care.

We will also evaluate whether the incidence of amputation and mortality, sickness absence, clinical measures (number of consultations, complications directly related to the foot ulcer as indicated by use of antibiotics), recurrence of a new foot ulcer (within 48 months), and patient-reported outcome measures (PROMs) are different for telemedicine follow-up in primary care compared with standard hospital outpatient care.

We conducted supplementary qualitative studies to provide a more comprehensive description and evaluation of the ongoing processes during the intervention. Individual interviews with patients in the intervention and control groups aimed to explore patient experiences with telemedicine follow-up or standard outpatient care delivered in the DiaFOTo trial. Focus group interviews with health care professionals either working in primary care or in specialist care delivering the intervention aimed to explore health care professional experiences when they adopt this new technology in caring for patients with diabetes foot ulcers.

## Methods

### Trial Design

**Figure 1:**
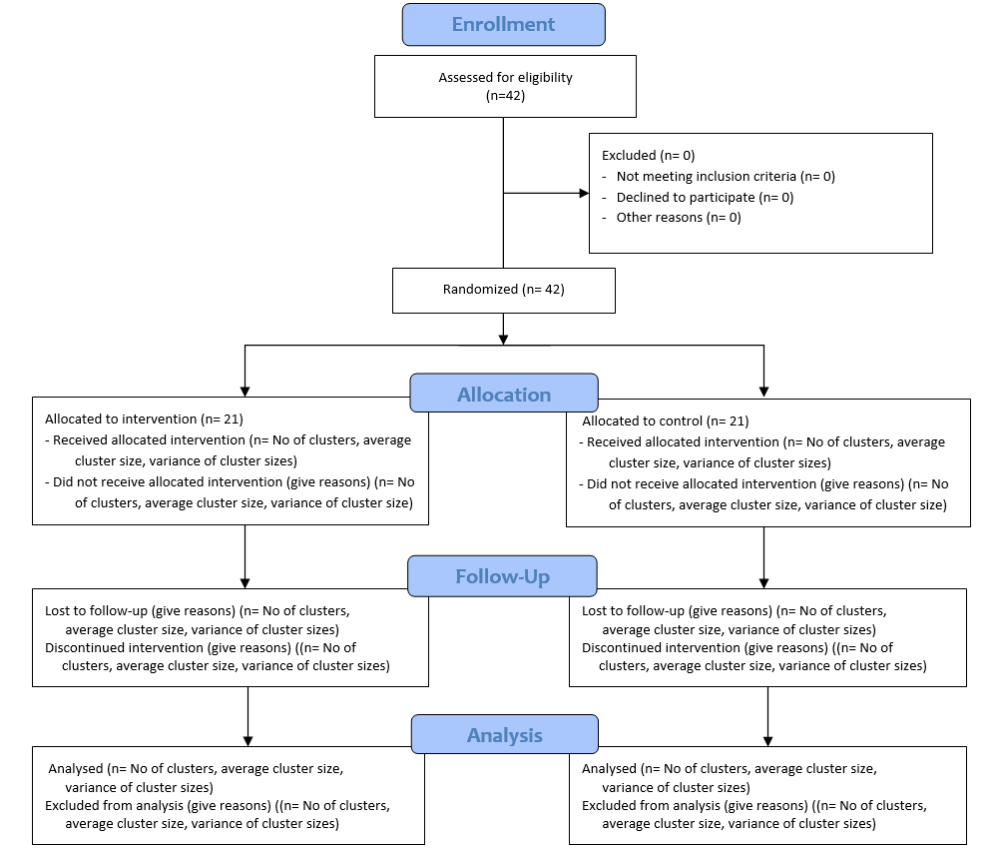
Flow diagram of clusters and patients in the cluster randomized controlled noninferiority trial, DiaFOTo.

In this pragmatic randomized controlled trial (RCT) [18], we evaluate the effectiveness of the intervention on patient health using the Model for Assessment of Telemedicine criteria [19] and the complex intervention framework developed by the Medical Research Council in the United Kingdom [20]. We used a noninferiority parallel cluster design. The flow and attrition diagram is shown in Figure 1 and based on the CONSORT 2010 statement: extension to cluster randomized trials [21]. Reporting will adhere to the guidelines of the Consolidated Standards for Reporting Trials (CONSORT) [22]. The trial is in accordance with the CONSORT EHEALTH checklist [23]. The study has been registered with ClinicalTrials.gov [NCT01710774].

Quantitative data are being collected at the time of inclusion (baseline) after informed consent has been obtained and before randomization (t1), and the patient is monitored every second week until the foot ulcer has either healed or until the end of follow-up (maximum 12 months after baseline) (t2). Additional information will be retrieved 36 months after the initial follow-up period about the occurrence of new foot ulcers, amputation, or death. Maximum follow-up for each patient is therefore 48 months.

### Trial Population and Recruitment

We are currently including all patients with diabetes-related foot ulcers from the southern part of Rogaland County referred to the endocrinology unit of Stavanger University Hospital (Stavanger HF) between September 2012 and June 2016 at the initial visit to the clinic.

The trial includes patients with type 1 or type 2 diabetes if they are 20 years or older and present a new foot ulcer to the clinical site. A foot ulcer is defined as a skin lesion below the ankle on a diabetic foot. The exclusion criteria include (1) an ulcer on the same foot treated during the past 6 months in specialist health care (because chronic ulcers can interfere with the primary outcome in the study protocol); (2) mental disorders or cognitive impairment (including schizophrenia, other psychotic disorders, and dementia); (3) inability to complete questionnaires in Norwegian, or (4) life expectancy of less than 1 year. Patients are being assessed for primary diagnosis and treatment at the clinical site according to standard protocols based on national guidelines [3].

### Randomization and Blinding

The southern part of Rogaland County in the western part of Norway was divided into 26 clusters based on municipalities or districts within municipalities. These were matched in pairs according to population size and rural/urban characteristics. Within each of the 13 pairs, the two clusters were randomly allocated to intervention or standard treatment. The randomization sequences were generated by an independent person using SPSS version 21 statistical software (IBM Corp). All patients in each cluster/municipality are in the same treatment group. All participants are informed by the study nurse about the allocated type of treatment after enrollment in the study and after providing baseline data. The intervention is designed to evaluate a change in health service provision; therefore blinding of the intervention is not possible. The study staff monitors whether pictures are submitted as planned and follows up to ensure compliance. We also assess how closely the nurses follow the protocol. The outcome is assessed by health care professionals at the outpatient clinics, who are not blinded to study arm but are trained to follow and assess wounds using a standardized protocol. The numbers of patients who do not meet the inclusion criteria, decline to participate, or drop out of the trial are recorded by age and gender.

### Development of the Intervention

The intervention consists of a Web-based ulcer record to facilitate asynchronous communication between primary and specialist health care and includes a database and an application to communicate images and text between participants. The Web-based ulcer record (Dansk Telemedicin AS) has been adapted to Norwegian legislation and is described in a previous publication [24]. We carried out a preliminary project in spring 2011 to develop and adjust the telemedicine tools to patients with diabetes-related foot ulcers receiving care in the community. In a pilot project between autumn 2011 and spring 2012, the data collection forms and telemedicine tools were tested on five patients. A standard procedure protocol was developed in brochure form to ensure that photographic documentation of ulcers was taken at optimal resolution, with adequate lighting and good contrast, from specified angles. The brochure also included instructions for use of the Web-based ulcer record, the integrated infrastructure, and legal and data security aspects.

### Telemedicine Intervention

**Figure 2:**
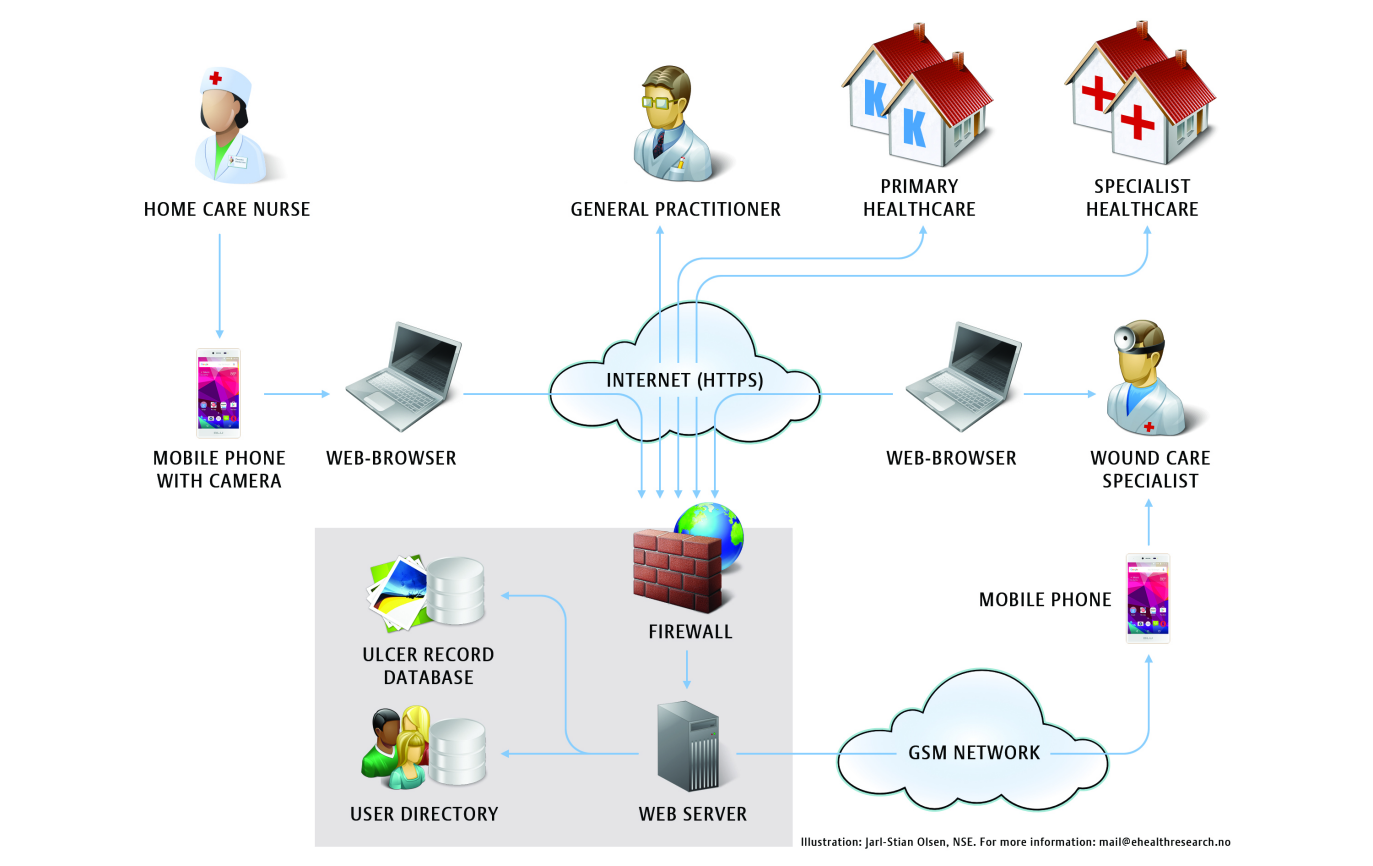
Diagram illustrating the general use of the telemedicine tool.

Patients in the intervention group receive telemedicine follow-up care in the community. The key ingredient in the intervention is the close integration between health care levels. The intervention is facilitated by the use of an interactive wound platform consisting of a Web-based ulcer record combined with a mobile phone, enabling counseling and communication between nurses in the community and specialist health care. Foot ulcer data including images are sent by mobile phone to the Web-based ulcer record for asynchronous consultation with specialist health care. Images are recorded throughout the trial, stored in the Web-based ulcer record, and transferred encrypted to a server. General practitioners in the intervention group can get access to the Web-based ulcer platform if required (Figure 2).

The nurses are trained to use the Web-based ulcer record and mobile phone using written information. Individual teaching and training of the nursing staff in primary care is offered at the specialist clinic or in primary care to secure equivalent and competent handling of patients. The diabetes specialist nurse and/or podiatrist in the multidisciplinary project team provide the latter. The written information also includes a section defining the delegation of responsibility at each level of health care providers with respect to the treatment of diabetic ulcers. Follow-up procedures are set up for each participant. The nurses review the images at the Web-based ulcer record and discuss them with the specialists if there is any uncertainty. Nurses in the specialist health care communicate with community care nurses at least once a week. In addition, nurses in specialist health care can check and contact community nurses on an ongoing basis. Discussions are mainly about wound care but could also include earlier referral if required. The home care nurse or general practice nurse will initiate contact with specialist health care regardless of prior agreements if the ulcers do not improve or get worse.

We have not made important changes to the intervention or control arm during the trial. Functionality has been evaluated every year and minor adjustments have been performed; however, this has not involved system failures/downtimes, etc. We have used the same photo documentation (smartphone/camera) throughout the project.

### Control Group

The control group receives standard hospital outpatient consultations with health care professionals at the endocrinology unit of Stavanger University Hospital. The medical treatment given to the control and intervention group is based on the same procedures. Foot ulcer data including images are recorded throughout the trial but only at the outpatient clinic. Like in the intervention group, the data are stored in the Web-based ulcer record and transferred in encrypted form to the same server.

### Evaluation Measures

The trial includes self-reported questionnaire data and information collected from the electronic patient journal and the clinical diabetes system at the outpatient clinic. The quantitative measures in our trial are described in detail below.

#### Primary Outcome Measure

The primary outcome is healing time measured from the time the person presents the ulcer at the clinical site (included in the trial) until the foot ulcer is healed.

#### Secondary Outcome Measures

Time to amputation (foot, below, through, or above the knee) and death during the patient follow-up period as well as self-reported questionnaire data will be used as secondary outcome measures in this trial. This questionnaire package will include standardized instruments measuring patient-reported outcomes as problem areas in diabetes, quality of life, symptoms of anxiety and depression, and satisfaction with treatment (see Table 1 for details).

**Table 1:** Summary of measures.

Outcome		Data collection instrument	Time points^a^
**Primary outcome**			
	Healing time	Time to healing. Data from electronic medical journals at the clinical sites.	t2
**Secondary outcomes**			
	Amputation (before healing)	Time to amputation. Data from electronic medical journals at the clinical sites.	t2
	Death (before healing)	Time to death. Data from electronic medical journals at the clinical sites.	t2
	Well-being during the previous 2 weeks [25-27]	The World Health Organization well-being index (WHO-5). Scale 0-5; higher scores indicating greater emotional distress.	t1, t2
	Symptoms of anxiety and depression during the past week [28-30]	Hospital Anxiety and Depression Scale (HADS). Two subscales, 0-3 (scored 0-21); higher scores indicate more symptoms.	t1, t2
	Diabetes-related problem areas [31-33]	Problem Areas in Diabetes (PAID). Scale 0-4 (scored 0-100); higher scores indicate more problems.	t1, t2
	Impact of diabetic peripheral neuropathy and foot ulcers on patient’s quality of life [34,35]	Neuropathy- and Foot Ulcer–Specific Quality of Life Instrument (NeuroQoL). Five subscales 1-5; higher scores indicate lower quality of life.	t1, t2
	Health status reflecting an individual’s subjective perception of health conditions [36-38]	Perceived health (or self-rated health). Scale 1-4; higher scores indicate better perceived health.	t1, t2
	Health-related problems and health related quality of life [39,40]	Euro-Qol (EQ-5D-5L). Scale 1-5; 1 represents “no problem.” Overall health, VAS-Scale, 0-100, higher scores indicate better health.	t1, t2
	Patient experiences [41]	Nordic Patient Experiences Questionnaire.	t1, t2
	The occurrence of new foot ulcers and amputation (after the initial follow-up period)	Data from electronic medical journals at the clinical sites.	t3
	Sickness absence	Norwegian sick leave registry (FD-Trygd registry).	t4
	Death (after the initial follow-up period)	Time to death (months). Cause of death registry.	t4
**Other measures**			
	Demographic characteristics (age, sex, ethnicity, education, cohabitation, marital status, working status and smoking, travel distance to hospital)	Patient questionnaire.	t1
	Clinical data related to diabetes and diabetes foot ulcer	Data from electronic medical journals at the clinical sites.	t1, t2,
	Consultations	Number in specialist care and primary care.	t2
	Wound classification [42,43]	University of Texas Diabetic Wound Classification System. Higher grade classified increasing wound depth (0-3). Higher stage classified the presence of infection and/or ischemia (A-D).	t1, t2

^a^t1: baseline assessment, t2: end of the initial follow-up period, t3: 36 months after end of the initial follow-up period, t4: will be merged with registry data after the trial is closed.

Long-term data on the time elapsing before a new foot ulcer appears and the incidence of amputation will be collected for 36 months after the initial follow-up period from the electronic patient journal at the clinical sites. Information on death (date and cause) will be retrieved from the Norwegian Cause of Death Registry.

#### Other Measures

We collect self-reported demographic data on age, sex, ethnicity, education, cohabitation, marital status, working status, and smoking status of the participant. In addition, clinical data are collected. For all participants, we store a picture and measure the wound area in the Web-based ulcer record system at the time of inclusion in the trial, after 8 weeks, and at the end of follow-up. The foot ulcers are classified according to the University of Texas Diabetic Wound Classification System [42], which combines grade and stage, is descriptive, and predicts clinical outcomes well (risk of amputation and healing time) [43]. At baseline, we collect data on blood pressure and measurements of neuropathy. Biological data include hemoglobin A_1c_ concentration and measurements of renal function (serum-creatinine, glomerular filtration rate, and microalbuminuria). Data from the medical records include type of diabetes, onset of diabetes, microvascular complications (retinopathy, neuropathy, and nephropathy) and macrovascular complications (myocardial infarction, stroke, claudication, and angina pectoris). Furthermore, we calculate the number of consultations, both in specialist health care and primary care.

### Sample Size

A statistical power analysis based on the primary outcome measure (healing time) was performed to decide the number of participants to be included using PASS sample size software, version 11 (NCSS, LLC). We powered the study to detect a difference in mean healing time larger than the selected noninferiority margin of 1.5 months [44,45], assuming 80% power, a significance level of .025, and a standard deviation of 3.6 months [46]. The analysis showed that on an individual level, a total sample size of 184 is needed. Considering an intraclass correlation coefficient of .02 and an average cluster size of 10 participants (design effect of 1.18), this number increased to 217 participants. As we expect an attrition rate of 5%, we aim to include 114 patients in each treatment group.

### Statistical Analysis

We will report descriptive statistics of baseline characteristics for the treatments groups including means and standard deviations (medians and interquartile ranges for continuous variables and numbers and percentages for categorical variables).

Noninferiority of the telemedicine intervention will be confirmed if the lower limit of the 95% confidence interval for the mean difference in healing time is less than 1.5 months. To account for correlated data introduced by the study design, we will apply the linear mixed-effects model [47] for this analysis. In addition, we will analyze group differences in time to healing with a proportional hazards model with adjustment of the standard error for clustered observations. Secondary outcome measures will be analyzed with superiority hypothesis tests. These tests will be based on either (generalized) mixed-effects models [47] or proportional hazards models (cluster) depending on the type of the outcome measure investigated. Estimated effect measures (absolute and relative) will be presented with 95% confidence intervals and *P* values.

Because our trial has a maximum follow-up period of 48 months, we expect some dropouts. We will perform intention-to-treat analyses and additional analyses based on the participants who actually participated actively in our trial during the 12-month follow-up period.

### Qualitative Substudies

#### Patient Experiences

Some participants with diabetic foot ulcers receiving either the telemedicine follow-up in primary care in collaboration with outpatient specialist care or standard outpatient care were individually interviewed to explore their experiences with telemedicine follow-up or standard outpatient care delivered in the DiaFOTo trial. Interviewees were selected to ensure a diverse sample in terms of group (intervention vs control), age, gender, marital status, setting, and comorbid diseases. The study nurses at the clinical sites organized recruitment of the patients. Patients were included if their foot ulcers had healed or after the intervention was completed.

Data were collected using individual semistructured interviews. The interview guide contained eight overall topics with subthemes for the intervention group and seven overall topics with subthemes for the control group. The topics were similar for both groups, except the control group were not asked questions related to the telemedicine equipment and the health care professional’s attitudes on using images in wound care (Textbox 1).

**Main topics in the interview guide (patient experience).**

Intervention group

- Patient experience with the foot ulcer and what he/she did when he/she discovered the ulcer
- Patient experience of receiving telemedicine treatment and follow-up from the home care nurse
- Patient experience of being followed up in specialist health care
- Patient experience of being involved in wound management and decisions that concerned his/her treatment
- Patient experience with health care professional use of the telemedicine equipment and health care professional’s attitude on using images in wound care
- Patient-observed telemedicine collaboration between the home care nurse and specialist health care service during follow-up
- Patient perception of whether he/she takes more responsibility for his/her own health
- Patient perception of the most important task home care nurses and experts at the outpatient clinic have in treatment and care of patients with diabetic foot ulcers

Control group

- Patient experience with the foot ulcer and what he/she did when he/she discovered the ulcer
- Patient experience of receiving traditional treatment and follow-up from the home care nurse
- Patient experience of being followed up in specialist health care
- Patient experience of being involved in wound management and decisions that concerned his/her treatment
- Patient-observed collaboration between the home care nurse or general practitioner and specialist health care services during follow-up
- Patient perception of whether he/she takes more responsibility for his/her own health
- Patient perception of the most important task home care nurses and experts at the outpatient clinic have in treatment and care of patients with diabetic foot ulcers

Data were collected and interviews carried out until saturation was achieved, in line with recommendations existing for qualitative research [48]. Transcribed interview text was analyzed by developing codes, grouping similar codes together in larger groups, and exploring these for patterns in terms of similarities and differences. As relationships became apparent, we interpreted them from a clinical and theoretical perspective. Several researchers were involved to support reflexivity of researchers throughout all phases of the qualitative study. Researchers analyzed data separately first and then compared and contrasted their analyses to reach a consensus on main themes and subthemes. Interpretive description was used as a strategy in this study. This approach to qualitative knowledge development for applied clinical fields aims to produce new knowledge and a contextual understanding that can be put to direct applied use when implementing the intervention in future clinical practice [49,50].

#### Health Care Professional Experiences

Interpretive description was also used as a research strategy for this study. Health care professional experiences with the intervention were explored and Donabedian’s framework was used to structure essential components of health services to be addressed in the study [51,52]. Information on health care professional experiences was collected through focus group interviews among those working in primary care or in specialist care delivering the intervention. We mixed different health care professions within their own working context so that different perspectives within their context could be explored and discussed. The focus groups were conducted by a moderator and comoderator among health care professionals in the initial stages of introducing telemedicine in their work. The semistructured interview guide covered topics related to our study aim (Textbox 2).

**Main topics in the interview guide (health care professional experience).**

- Participant experience using telemedicine and how it was organized where they work
- Participant experience using telemedicine as a new tool in documentation and communication
- Participant experience of communication and collaboration between outpatient clinic (physicians, nurses, and foot therapists) and nurses in home care through telecommunication and among professions
- Participant perception of changes in competence in caring for people with diabetes foot ulcers during the intervention
- Participant perception of changes in job satisfaction while using telemedicine

### Ethical Considerations

All participants receive written information about the project and its aims with a description of the procedures of the project before inclusion. We inform participants that their privacy will be protected and all data will be coded and processed anonymously to protect confidentiality and that they can withdraw from the project at any time without this affecting their treatment. All patients are required to provide written informed consent before participation. We do not believe that the project has any negative effects for the patients involved that would raise problematic or specific ethical issues. Completing the questionnaire might be a burden, but we do not consider this burden to exceed the potential new knowledge and evidence the study will produce. The project is approved by the Western Norway Regional Committee for Medical and Health Research Ethics (2011/1609), which also has given approval for merging our data with the Norwegian cause of death and sick leave registries.

The Norwegian Centre for Integrated Care and Telemedicine lent its expertise in data security and legal aspects to the planning of this project. In line with Norwegian legislation and security services, data controller agreements were performed between all parties. We performed risk assessment analyses after the pilot project and after years 1 and 2 of the trial. Information about the project has been disseminated in collaboration meetings at different administrative levels in the involved municipalities. Qualitative substudies were performed to include the perspective of health care users. In addition, a representative from the Norwegian Diabetes Association is participating in the project group. We have established standardized procedures for transfer of data, security, and storage of data in collaboration with the Norwegian Centre for Integrated Care and Telemedicine.

## Results

The study has been successfully funded. The inclusion of patients started September 2012 from 26 municipalities or districts. Because recruitment goals were not met in the initial period, two more hospitals from the Western Norwegian Health Region have been included to meet sample size requirements. Patients from Sunnhordland County referred to the department of surgery at Stord Hospital (Helse Fonna HF) were included from September 2013 (6 districts), and patients from Hordaland County referred to the department of orthopedics or endocrinology unit at Haukeland University hospital (Helse Bergen HF) were included from November 2014 (10 districts), for a total of 42 districts before randomization (Figure 1). Furthermore, patient recruitment has been extended through June 2016. We expect to present results of the study in 2017.

Data collection in the qualitative substudies has been completed. Information on patient experiences was collected between March 2014 and May 2015 from 24 participants with diabetic foot ulcers receiving either the telemedicine follow-up in primary care in collaboration with outpatient specialist care or standard outpatient care. Information on health care professional experiences was collected through 10 focus group interviews from 7 home-based care services, 2 outpatient clinics, a medical center, and a nurse-led primary care clinic during 2014 and 2015 (n=43). Focus group interviews lasted from 70 to 90 minutes, included 3 to 7 health care professionals, and were audiotaped. The results of these studies are submitted and will be available in 2016.

## Discussion

This project will contribute to increased focus on integrated care and is in accordance with national strategies [12]. By transferring the follow-up care to the lowest effective service level, we anticipate that results of this trial will improve the motivation and awareness of health care professionals in the community to implement disease prevention measures. We believe that telemedicine can become a tool to raise the competence of nurses in the community and facilitate better communication and closer collaboration between health care levels, improving both foot ulcer care and general diabetes care. In this project, patients are not sending pictures directly to specialist health care services because it would be difficult for patients with diabetic foot ulcers to take pictures themselves due to location of the ulcers, frailty, age, and impaired vision. However, we expect that telemedicine-based ulcer follow-up can positively influence patient competence and involvement in diabetes self-management, including using preventive strategies to avoid or delay new foot ulcers. If the study finds evidence of positive health gains for the individuals with diabetes and contributes to a higher quality of care, this new model may be applicable to other hospital trusts and health care regions.

An important concern due to internal validity is whether the intervention is working similarly in all communities and within the community itself. The nurses in the community are trained by using written information. Individual teaching and training of the nursing staff in primary care is offered at the specialist clinic or in primary care to secure equivalent and competent management of patients. We have not made important changes to the intervention or control arm during the trial, and we used the same photo documentation (smartphone/camera) throughout the project. When designing the study we stressed the internal validity. The rationale for choosing a cluster-randomized trial was that classic randomization could threaten the internal validity because nurses in the municipalities would treat patients in both the intervention and control groups. During the study, we have used qualitative studies to explore in detail how this complex intervention is working from a patient and provider perspective. Results from the trial as well as results from the qualitative studies will be published in peer-reviewed journals to the international audit. All studies will emphasize internal and external validity in line with the Model for Assessment of Telemedicine criteria [19].

One of the concerns of this complex intervention study is whether the patients included in the trial are representative of the majority of patients with diabetic foot ulcers. Patients with more complex illness living in nursing homes or having mental problems and those having difficulties traveling to a hospital may benefit from this type of intervention the most but will be excluded from participation in the present trial due to their vulnerability. In addition, we excluded patients with a previous ulcer within 6 months of presentation since repeated chronic ulcers may interfere with the primary outcome. Therefore, our cohort does not fully reflect the total population with diabetic foot ulcers attending the participating clinics. To increase the number of patients included in the trial, the study is embedded in daily clinical practice at three clinical sites. This will contribute to increasing the external validity and generalizability of the results and thus make them more applicable to a realistic clinical setting.

We expect this project to provide evidence about alternative care pathways for the treatment of diabetic foot ulcers that may reduce the cost of health care services by delivering a larger proportion of services in municipal primary care. This study may also contribute to setting priorities for patient needs for flexible health services and enable more patients to be treated near their homes.

## Abbreviations

DiaFOTo: Diabetic Foot and Telemedicine Images Project
PROM: patient-reported outcome measure
RCT: randomized controlled trial
CONSORT: Consolidated Standards for Reporting Trials
